# Tinea Incognito Caused by *Microsporum* spp. Mimicking Subacute Cutaneous Lupus Erythematosus—Case Report

**DOI:** 10.3390/jof11070530

**Published:** 2025-07-17

**Authors:** Marta Kasprowicz-Furmańczyk, Agnieszka Owczarczyk-Saczonek

**Affiliations:** The Department and Clinic of Dermatology, Sexually Transmitted Diseases and Clinical Immunology, Faculty of Medicine, Collegium Medicum, University of Warmia and Mazury, 10-719 Olsztyn, Poland

**Keywords:** tinea incognito, trichoscopy, subacute cutaneous lupus erythematosus, *Microsporum* spp., glucocorticosteroids

## Abstract

Tinea incognito is an incorrectly diagnosed form of fungal infection due to a changed clinical picture as a result of systemic or topical corticosteroids or even local immunomodulators. This type of skin lesion is most often located on the trunk but can affect any part of the body. We present a case report of 76-year-old woman with a history of systemic lupus erythematosus who was admitted to hospital because of extensive, painful, and burning erythematous and papular lesions in an annular pattern, covered with a thick, yellow crust, located on the scalp and neck. The skin lesions were accompanied by extensive hair loss. The patient had previously undergone intensified treatment of the underlying disease due to the exacerbation of skin lesions of a subacute cutaneous lupus erythematosus type. A suspicion of tinea incognito was raised, and direct mycological examination and culture confirmed the presence of dermatophytes (*Microsporum* spp.). Tinea incognito can be difficult to diagnose because the clinical picture is relatively nonspecific and can mimic other dermatoses, such as subacute lupus erythematosus. Therefore, in doubtful cases it is necessary to perform a direct test and culture for fungal infection, especially before initiating treatment with glucocorticosteroids and other immunosuppressive agents.

## 1. Introduction

Clinically, tinea corporis manifests itself as characteristic, round, erythematous-scaling lesions, clearly demarcated from healthy skin. Papules, pustules, or vesicles may occur at the edges of the lesions. The lesions spread centrifugally. The disease is often accompanied by persistent pruritus [1]. A clinical and diagnostic challenge in the case of tinea corporis and tinea capitis is the recognition of the disease in patients previously treated with immunosuppressive drugs such as glucocorticorticosteroids [2]. Fungal culture remains one the most important methods of confirming dermatophytosis, especially in cases where the diagnosis is uncertain, other test results are inconclusive, or the infection is extensive and resistant to treatment. A very useful and reliable method is real-time PCR including species determination. To perform the analysis, DNA isolation from infected scales or hairs is sufficient. Species determination can be carried out immediately, without time delay, while culturing requires weeks of time [3]. Other diagnostic options are dermoscopy and examination of skin scrapings from the active lesion under a microscope using a potassium hydroxide preparation. The first-line treatment for tinea corporis typically involves topical antifungal agents. However, systemic antifungal therapy is recommended when lesions are numerous, recurrent, chronic, or if there is no improvement after topical treatment [4].

Tinea incognito was first described by Ive and Marks in 1968 [5]. This is a dermatophyte infection exacerbated by inappropriate immunosuppressive treatment (most often with glucocorticosteroids), which, due to its non-specific clinical picture, can imitate many different dermatological diseases such as eczema, psoriasis, or lupus erythematosus [5,6]. We present a case of a patient with systemic lupus erythematosus treated chronically with glucocorticosteroids who developed lesions clinically resembling subacute cutaneous lupus erythematosus (SCLE). After performing a direct examination, culture, and trichoscopy, a diagnosis of tinea incognito caused by *Microsporum* spp. was made.

## 2. Case Description

A 76-year-old patient was urgently admitted to the Dermatology Clinic due to extensive erythematous and papular lesions on the scalp and neck, accompanied by alopecia in the scalp and severe subjective symptoms such as pain, itching, and burning. The patient was diagnosed with systemic lupus erythematosus in 1990. Due to disease exacerbation, since 2024, she has been treated with oral prednisone (15 mg/day), mycophenolate mofetil (1000 mg/day), hydroxychloroquine (200 mg/day), and topical tacrolimus with good therapeutic effects. In 2025, the patient was hospitalized three times, two times in the Dermatology Clinic and once in the Rheumatology Clinic.

**Figure 1:**
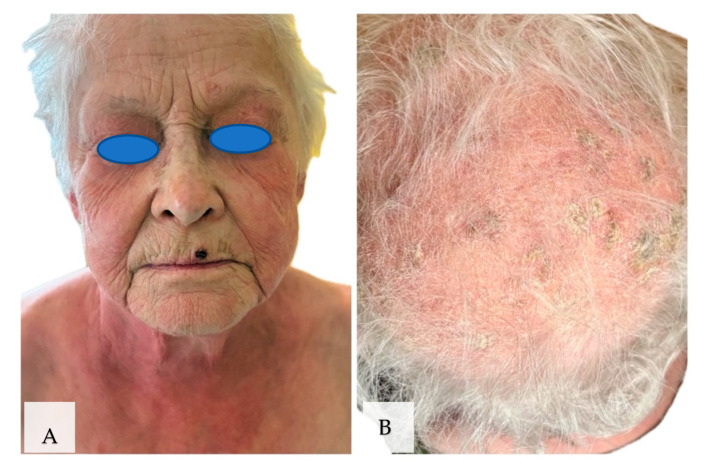
Appearance of lesions during the first hospitalization. (**A**) Well-demarcated erythematous-infiltrative lesions located on the face, eyelids, neck, and chest. (**B**) Erythema and numerous erosive lesions on the scalp covered by scales.

On 25 February 2025–27 February 2025, the patient was admitted to the Dermatology Clinic due to the appearance of well-demarcated erythematous and infiltrative lesions on the skin located on the neckline, back, and upper limbs. Erythematous lesions were also found on the face and eyelids (Figure 1A). There was erythema and numerous erosive lesions on the scalp covered by yellowish scales (Figure 1B). During hospitalization, laboratory tests were performed without finding any significant deviations. Anti-nuclear antibodies were positive in titer 1:640; in immunoblotting, there were positive RNP/Sm +++, RNP70 ++, and RNP A ++. Direct mycological examination of the lesions on the scalp was performed, without finding the presence of fungi. Scrapings from the scalp were taken for culture, also with negative results. A throat swab revealed *Candida albicans*, and treatment with fluconazole 100 mg/day was recommended. The prednisone dose was increased to 20 mg/day. During hospitalization, the patient was consulted with a rheumatologist. Hospitalization in the Rheumatology Clinic was planned to modify the general treatment due to the exacerbation of the disease.

On 28 April 2025, the patient was admitted to the Rheumatology Clinic. Laboratory tests did not reveal any significant deviations, but there was a further exacerbation of erythematous–exfoliative lesions on the scalp, and generalized alopecia occurred. During hospitalization, the patient was given a pulse of metyloprednizolon (IV 3 × 1000 mg), mycophenolate mofetil treatment was discontinued, and MTX at a dose of 15mg was included in the treatment. A dermatological consultation was held, during which a repeated mycological examination of the scalp was ordered. Scrapings from the scalp were taken onto a culture medium (malt/extract/corn meal/DTM–Taplin), and the other sample was sent to the microbiology laboratory. The patient was discharged from the clinic on 5 May 2025 in good general condition.

**Figure 2:**
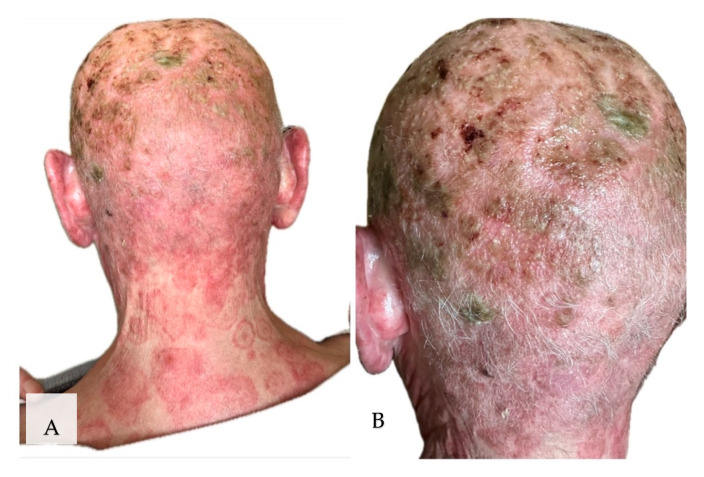
Appearance of lesions during the third hospitalization. (**A**) Numerous erythematous and papular lesions with an annular pattern, spreading centrifugally on the neck and nape area. (**B**) Diffuse dark red infiltrative lesions covered with thick yellow scales, occupying the entire scalp. Almost complete hair loss.

**Figure 3:**
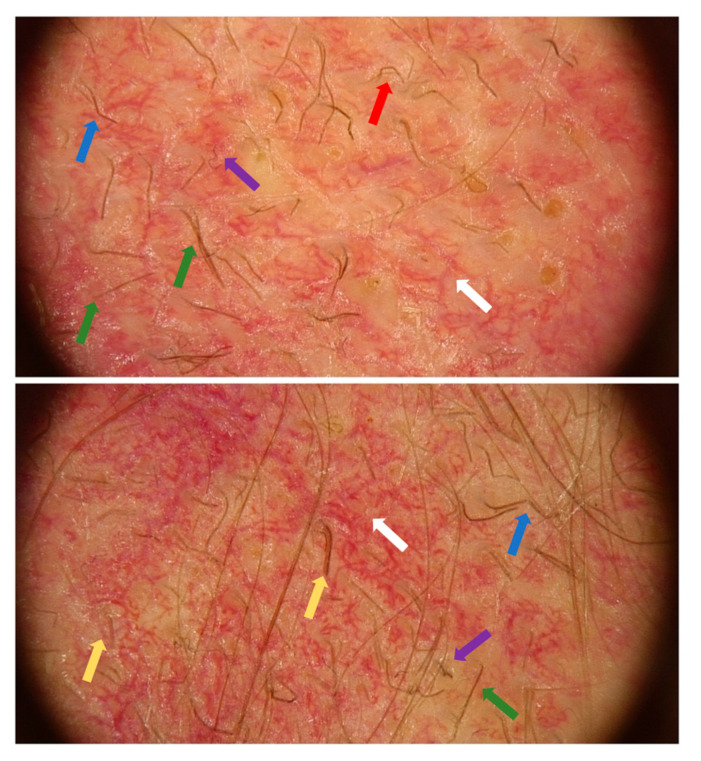
Trichoscopic examination of the scalp revealed thick arborizing blood vessels and structureless milky-red areas (white arrow), which are characteristic for lupus erythematosus, and comma hairs (red arrow), zigzag hairs (blue arrow), Morse-code-like hairs (green arrow), corkscrew hairs (purple arrow), and block hairs (yellow arrow), which are characteristic for tinea.

**Figure 4:**
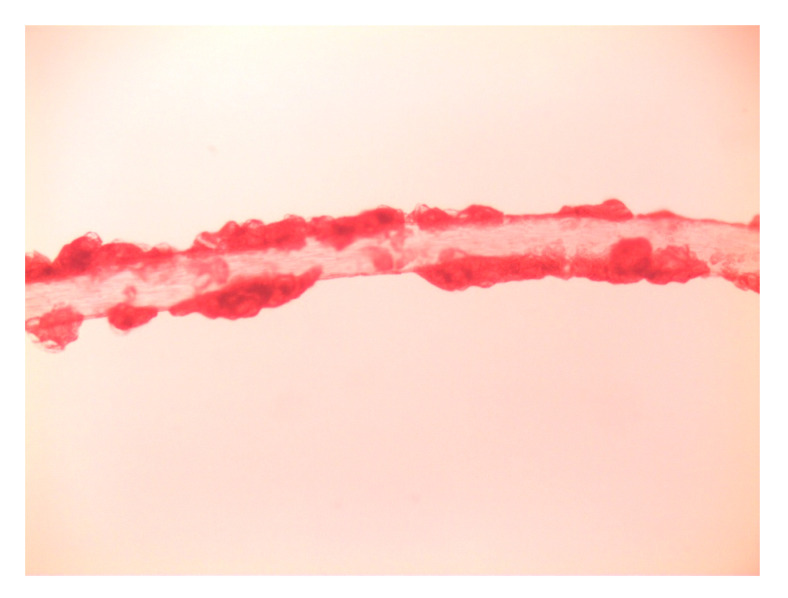
Direct microscopic examination of hair. Ectothrix. (20× magnification).

**Figure 5:**
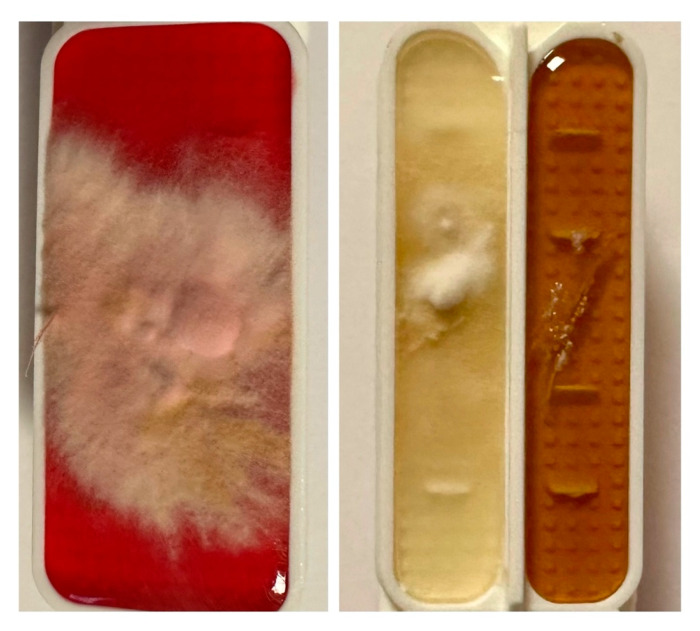
Culture grown from the skin lesion scrapings. Cottony, powdery, cream-beige colonies.

**Figure 6:**
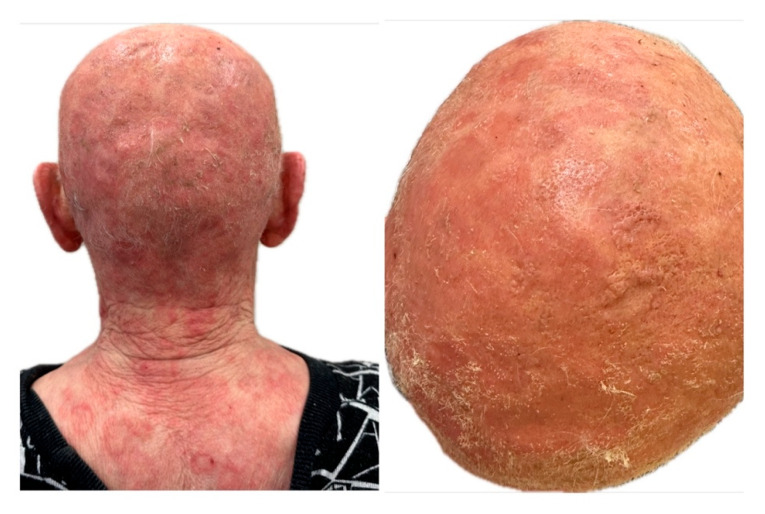
Local condition after 3 days of therapy with terbinafine.

On 7 May 2025, the patient reported urgently to the Dermatology Clinic due to burning and stinging pain in the scalp area with a VAS pain intensity of 10 points. Physical examination on admission revealed diffuse dark red infiltrative lesions covered with thick yellow scales occupying the entire scalp. There was almost complete hair loss. In the neck and nape area, there were numerous erythematous and papular lesions with an annular pattern, spreading centrifugally (Figure 2A,B). Trichoscopy revealed thick arborizing blood vessels and structureless milky-red areas but also numerous comma hairs, zigzag hairs, Morse-code-like hairs, block hairs, and corkscrew hairs (Figure 3). Laboratory tests were taken, and no significant abnormalities were found. A direct mycological examination was performed, revealing the extra-hair arrangement of spores (ectothrix). For direct examination of hair, samples were placed on a glass slide, treated with 10% KOH and examined microscopically to identify the type of invasion. (Figure 4). A few days after collection of samples from the scalp during hospitalization in Rheumatology Department, the growth of dermatophyte colonies was observed, with a characteristic change in the color of the medium to red (Figure 5). Terbinafine was empirically introduced into the treatment at a dose of 250 mg daily with a significant improvement in the local skin condition (Figure 6). After 4 weeks of collection, the results from the microbiology laboratory were received, which diagnosed a *Microsporum* spp. infection.

## 3. Discussion

Tinea incognito is a dermatophyte fungal infection in which systemic or topical steroids can lead to the spread of infection, causing skin lesions in the course of mycosis to look atypical [7]. They may have a less scaly appearance, less raised edges, are more pustular in nature, but are often severely irritated and inflamed. Initial improvement of the local condition after the use of immunosuppressive drugs with the disappearance of subjective symptoms is characteristic, whereas after discontinuation of treatment, the disease exacerbates with a rapid peripheral spread of lesions [8]. Tinea incognito occurs with similar frequency in all age groups, except during infancy and in the elderly over 75 years of age [9]. The most commonly identified dermatophytes are *Trichophyton rubrum* (anthropophilic), *Trichophyton mentagrophytes*, and *Microsporum canis* (zoophilic) [10]. Studies indicate that the most affected area was the torso, followed by the face [10,11]. One study found that one third of patients also had fungal disease involving distant areas of the body, such as the feet and nails [11]. This suggests that autoinoculation to any other part of the body is possible, especially if the patient has been previously treated with immunosuppressive drugs.

Treatment of tinea incognito is standard and does not cause any difficulties; anti-inflammatory drugs should be discontinued, and oral or topical antifungal therapy should be initiated. However, it can be difficult to make the correct diagnosis, because tinea incognito mimics numerous dermatoses. Romano et al. retrospectively reviewed 200 cases of patients with this disease. The lesions most often mimicked eczema, bacterial infections, and discoid lupus erythematosus. Less frequently described are rosacea, seborrheic dermatitis, lichen planus, pityriasis rosea Gibert, and psoriasis. Tinea incognito can often be misinterpreted as an exacerbation of a pre-existing chronic disease [12].

When tinea incognito of the scalp is suspected, in addition to direct examination and culture, trichoscopy may be a helpful diagnostic test. The characteristic symptoms of scalp mycosis in dermatoscopy are comma hairs, corkscrew hairs, Morse-code-like hairs, block hairs, I-hairs, and zigzag hairs [13]. An interesting analysis of the use of trichoscopy in tinea capitis was made by Waśkiel-Burnat et al., who showed that trichoscopy can be useful in establishing the primary diagnosis of tinea capitis and is a useful method in differentiating *Microsporum* spp. from *Trichophyton* spp. tinea capitis [13]. In the case of *Trichophyton* spp. infection, corkscrew hairs were observed more frequently than in *Microsporum* spp. tinea capitis. In contrast, Morse-code-like hairs, zigzag hairs, bent hairs, and scattered scaling of the scalp were observed only in patients with *Microsporum* tinea capitis. There was no significant difference in the frequency of broken hairs, black dots, and comma hairs between the two types of dermatophytosis [13].

In our patient’s case, characteristic features of chronic systemic lupus erythematosus can be observed, such as thick arborizing blood vessels [14] and structureless milky-red areas. However, upon closer examination, features characteristic of tinea capitis can be seen, such as comma hairs, zigzag hairs, and Morse-code-like hairs, which indicate *Microsporum* spp. Infection [13] (Figure 3).

The case presented in the article confirms how important a part of the diagnostic process are direct mycological examination and culture, which help to make the correct diagnosis and, consequently, implement the correct treatment. In the case of lesions located on the scalp, trichoscopic examination may be valuable. A lack of time due to the overload of the healthcare system and a lack of access to direct mycological examination in general practice can lead to misdiagnosis and unnecessary treatment with glucocorticosteroids, leading to the occurrence of tinea incognito [15]. Another significant problem is the wide availability of glucocorticosteroids and other immunosuppressive drugs without a prescription, as well as the recommendation of drugs combining steroids with antibacterial and/or antifungal drugs without making an accurate diagnosis by physicians who are not dermatologists [2]. Both topical and systemic glucocorticosteroids inhibit the body’s natural defenses against dermatophytes, which promotes the development of superficial fungal infections [2]. Frequent skin examinations are necessary in patients with chronic dermatoses taking immunosuppressive drugs. Always, if lesions do not improve with classical therapies, the diagnosis should be verified, and tinea incognito should be considered [16].

## 4. Summary

Tinea incognito is an exacerbation of dermatophyte infection after improper systemic or local administration of glucocorticosteroids. It is a common problem observed in everyday medical practice, resulting from the wide availability of GKS preparations, often without a prescription. Tinea incognito can imitate many different dermatoses, causing diagnostic and therapeutic difficulties. The case we describe shows how important it is to monitor patients treated with chronic immunosuppressive drugs in everyday medical practice. The lack of therapeutic efficacy of classic therapies should always prompt verification of the previously made diagnosis.

## Data Availability

The data presented in this study are available at [1,2,4,5,6,7,8,9,10,11].
